# On the Secale Cornutum, Clavus, or Ergot of Rye

**Published:** 1825-08

**Authors:** Henry Davies


### ART. III.—*On the Secale Cornutum, Clavus, or Ergot of Rye.* By HENRY DAVIES, M.D.

[Concluded from page 7.]

CASE XII.—*Abortion.* Mrs. Page, aged twenty-two years, first pregnancy. March 12, 1821, at twelve at night, aborted a fœtus, apparently about the fifth month. The expulsion was followed by comparative ease, and she went to sleep. On being called to her at five o'clock in the morning, she was very anxiously sitting on the bed, over warm water. The parts were so confined, as to cause great pain on making an examination; and, on endeavouring to deliver the placenta, which could be just felt within the os uteri, she made so violent an outcry, and was so restless, that no further attempt was made. An infusion of secale (one drachm to three ounces of boiling water,) was given: it was not followed, as is usual before the birth of the child, by any effect. A second infusion, made from the same secale, was given about an hour after. At eight o'clock a domestic enema was given, and repeated at nine o'clock; which removed a considerable quantity of hard fæces, and caused a continued desire to go to stool. The placenta was expelled into the vagina; from thence removed by the finger. No hemorrhage or discharge followed. At ten o'clock she was left comfortable. Her recovery was in every respect good.

CASE XIII.—September 22, 1821.—Mrs. ——— was supposed to have aborted in the country; but, as she had borne many children, and was convinced that she was upwards of fourteen weeks' pregnant, she did not feel satisfied with that opinion; she chose to return to London, a distance of seventy miles. She was tolerably well for a day or two, except that she felt anxious. On the fourth day after her arrival, she discovered something in the vagina: this was a small funis, which, on being traced to its attachment, the bones of a fœtus were just perceptible within the os uteri, which was dilated to a small extent. A few hours after, the fœtus, with the funis, was delivered by some assistance. The operation gave but little pain; scarcely any discharge followed. The vagina was now clear, and not any thing could be felt at the os uteri, which was very high up, and soft. The uterus still contained something; and, as we now felt assured that what came from her in the country was merely a coagulum of blood, the placenta therefore must be left. This alarmed her much, as she had read in some book that death had ensued from this cause. Some medicine was given to quiet her; and two doses of castor-oil, and one or two enemas, were also administered between the delivery of the fœtus and the second day at noon, when a strong infusion of the secale, to the extent of three doses, was given. This did not produce the slightest effect in any one way.

On the day following, she was apparently quite well.

Early in the morning of the next day, (fourth from the delivery of the fœtus, and ninth from the date of the attack,) she was awoke by a sudden discharge of water from the vagina; followed, a quarter of an hour after, by hemorrhage to some extent: she fainted. Another fœtus was felt presenting: this was delivered with some difficulty, as the os uteri embraced the head, and descended with it. Shortly after, one, and then a second, placenta was delivered. She recovered well, and has since borne a child at the full time.

In other cases of abortion, which were protracted, the secale has been given, both in powder (one scruple doses,) and in infusion, with little or no apparent effect.

In cases where premature labour has been intended to be brought on, by detaching the membranes near the os uteri, or by puncturing them, it has not produced any thing like a proportionate effect, unless labour was already going on: then it has had some effect, but not equal to that procured in labour at the full period.

In flooding cases, it is more frequently thrown off the stomach by vomiting; and, as other means were also had recourse to, its effect cannot be spoken of decidedly.

In craniotomy cases, after the head has been broken down, and where the child was still firm and stationary, the secale has very much accelerated the delivery.

The secale has been said to be useful in amenorrhœa; but, as this disease is more frequently dependent on a disordered state of the system, than on deranged function of the uterus merely, it has not been often exhibited; and, in those cases where the general health has appeared re-established, and the menstrual secretion has not appeared, the secale given in infusion, of two drachms to eight ounces, in doses of one ounce and a half, or from five to ten grains of the powder, three times a-day, little more than slight uneasiness (and this is not very apparent,) has been produced by it. Larger or stronger doses, perhaps, might be more useful. It has been given in decoction, in the proportion of one ounce of the secale boiled in two pints of water to one; of which, one ounce and a half was given three times a-day, and it is said, where a strong infusion was employed, and persevered in for a month, that the results were beneficial.

*Cases of Uterine Tumor, in which the Secale was administered.*

CASE XIV.—Miss M—— had been for a considerable time suffering from uterine disease. There was a tumor completely filling the vagina, which was removed by ligature. Shortly after, the vagina was again filled with a similar tumor, portions of which came away in large flakey pieces, and in heart-shaped lumps, in appearance somewhat resembling the cerebellum. In order to induce the uterus to expel the whole into the vagina, it was agreed, in consultation with Dr. Merriman, to give the secale. The annexed is the report of its effects, as given by Mr. Langley, who superintended the case.

" I gave last night an aperient medicine, which had the effect of twice opening the bowels. Being called out to a labour early this morning, I was prevented giving the secale till one o'clock P.M. I divided one drachm, finely powdered, into three equal parts,—one of which I gave mixed in treacle. At twenty minutes after one, I was informed that the stomach had discharged its contents, and of course, I conjectured, the principal portion of the dose of secale. I now gave a saline effervescent draught, with a view of quieting the stomach. In half an hour another dose of the secale was given, which was retained.

" I must here observe that, in the short space of fifteen or twenty minutes from the period of exhibiting the first dose, pains, assuming the character of uterine contraction, came on, and have been increasing in violence, with all the concomitants of bearing down, frequent inclination to pass urine and fæces, &c. ever since; and now (nine o'clock) the third dose has been given. The pulse was 120 when I gave her the first dose, the same at five o'clock, and the same now. I have examined, and do not find any material difference in the situation of the tumor; but evidently perceive it pressing downwards into the vagina during the pains, of which there is very little intermission. Her mother gave the third dose during my absence; otherwise I should have hesitated, as the pain is now very poignant.

" A little before one o'clock in the morning, during a very severe bearing-down pain, and while the patient was sitting over hot water, a large lump came away. The pains continued throughout the night, but have remitted this morning at nine o'clock."

No more of the secale was exhibited for a day or two, when a similar effect was produced by it, and more of the tumor came down. Notwithstanding this, the tumor increased more rapidly in proportion as parts of it were removed; the use of the secale, therefore, was laid aside. What became of this

CASE XV.—Mrs. Dill had a tumor in the vagina coming down from the uterus, very much resembling the former case. The secale was given to her in scruple doses, in order to bring the substance more under command: its effects were very similar. The tumor was removed by ligature, and its re-appearance in this case seems arrested.

From the above cases, and those related in the works contained in the margin,* the secale evidently possesses peculiar powers in influencing the uterus in certain states; and this effect in these states is as much to be relied on as the action of emetics and purgatives, in cases where t[illegible]y may be required. There are, of course, certain indications and precautions as necessarily required for its exhibition, as in that of any other powerful agent. The beneficial effects resulting from the exhibition of any remedy depends on its timely application; but the advantages and disadvantages likely to result from the use of the secale, and the indications for its exhibition, will be mentioned in the sequel.

Where secale is indiscriminately exhibited, the contractions of the uterus are rendered unnecessarily violent. It has been said to cause the death of the child; but it will admit of a query whether more injury would be likely to result from strong pressure for half an hour, with little intermission, or from moderate continued pressure an indefinite length of time. There is little doubt that, if the strong action of the uterus is excited by the secale when the parts are in an undilated state, that here the violent pressure on the body of the child may, where it can neither advance nor retreat, in some instances occasion its death. This, however, cannot be attributed to the medicine, but to the untimely exhibition of it. And, further, when it is considered that the use of this medicine is only indicated in those cases which would be long protracted, it will not be thought that this unfortunate event happens more frequently than in similar cases where the secale is not used; and it should be borne in mind that the labour is terminated with infinitely less exhaustion and tediousness on the part of the patient.

It has been also said to cause retention of the placenta. The generality of cases detailed by no means bear out this assertion. Allowing, however, that this result may follow in some cases, from the rapid delivery of the child, surely such effect might be very much obviated by graduating the expulsion of the child, and following up the contraction of the uterus by moderate pressure being made on the abdomen, as so well pointed out in Dr. Ramsbottom's work.

* *London Medical and Physical Journal*, vol. xxxii. and xxxvi.; CHAPMAN'S *Materia Medica;* Dr. MERRIMAN *on Difficult Paturition; Philadelphia Journal of the Medical and Physical Sciences*, Nos. 15 and 17; EBERLE'S *Materia Medica; Dictionnaire Abrégé des Sciences Medicales.*

It is a medicine that may be liable to abuse, to gratify unjustifiable impatience on the part of the patient, or to shorten the attendance of the midwife,—in preternatural presentation, and in deformity or contraction of the pelvis.

The powers of the secale appear to be wholly confined to its action on the uterine fibres, when that viscus is in a state of enlargement. It does not appear, however, to bring on labour, or cause action, in the first instance; but to increase the action when weak, or renew it when suspended. The uterus is thus made to press closely upon any substance whatever within its cavity. The healthy unimpregnated uterus, having nothing within it, will therefore not be affected by the secale: hence it is said not to restrain menorrhagia, as the uterus is then little more than its usual size.

Of the secale, when the fœtus has been some time dead, and putrefaction to any extent taken place, it has been observed, that the remedy is altogether inert. Is this the reason that it has so little effect in cases of abortion?

In no case has it been followed by hemorrhage; and, in natural labour, where hemorrhage does occur, it has been said to be given with great advantage. It is particularly recommended as a preventive of hemorrhage to patients who have formerly suffered from flooding subsequent to the birth of the child, from unfavourable separation of the placenta. In this case, an efficient dose, from one scruple to half a drachm, finely powdered, should be given, a quarter of an hour previous to the probable delivery of the child.

The following indications may be laid down for its exhibition. —The secale ought never to be given where there is any natural defect, either in the pelvis or soft parts, capable of producing a powerful obstacle to the expulsion of the child. Neither in those cases where the neck of the uterus is hard, swollen, or painful; in short, where there is rigidity of the parts: and, generally, where the abstraction of blood is indicated, this medicine is improper. The labour should have made some progress; the parts should be well lubricated with the natural mucus; the uterine orifice fully dilated, and all the soft parts prepared for delivery. The practitioner should, by careful examination, &c. be assured that delivery is retarded only by defective action of the womb. The presentation should be natural, and the child so situated that delivery can be effected by the efforts of the uterus.*

If the patient is very much fatigued and feeble, it should not

---

* It may be observed, that in some cases, where the pelvis was a little confined, and where the head was not sufficiently low down for the application of the common forceps, the secale has been successfully used, and the child delivered with the forceps: very great discretion, however, in these cases is required.

be given till she is refreshed and recruited, by suitable nourishment or medicine, lest the exertion it occasions should be more than she can bear.

The secale may be given in infusion or finely powdered; in either form its effects are similar: the powder, perhaps, acts more speedily; but, where there is a disposition to sickness, the infusion is better borne on the stomach. It would appear to be more effective when given in a large dose at once, than in small doses frequently repeated, which rather tend to fatigue the uterus unnecessarily. It has been suggested by Dr. Begeschi, in the Annali Universali, that the dose should be varied according to the strength and constitution of the patient; her temperament, age, and state of health at the time; whether she be more or less fatigued; and whether the membranes be whole or ruptured. The infusion of one drachm in three ounces of boiling water for twenty minutes, is usually an efficient dose, or one scruple finely powdered. As in some cases the effect does not always follow, the same dose may then be repeated in the space of an hour.

